# Disentangling the Bidirectional Relationships Across the Corporate Sustainable Development Indicators

**DOI:** 10.1007/s11205-022-02899-5

**Published:** 2022-02-28

**Authors:** Khine Kyaw, Julio Pindado, Chabela de-la-Torre

**Affiliations:** 1 NTNU Business School, Norwegian University of Science and Technology, 7030 Trondheim, Norway; 2 IME, University of Salamanca, Campus Miguel Unamuno, Edificio FES, 37007 Salamanca, Spain; 3 Leeds University Business School, University of Leeds, Leeds, LS2 9JT UK

**Keywords:** Corporate sustainable development, Economic indicator, Environmental indicator, Social indicator, Governance indicator, Dynamic panel data

## Abstract

This study disentangles the relationships that exist between the four indicators of corporate sustainable development: economic, environmental, social, and governance. We account for the potential bidirectionality of the relationships, control for the dynamic nature of the sustainability process, and address the endogeneity problem to appropriately analyze the sustainability process. We estimated a panel data from 734 U.S. companies from 2004 through 2016 by using the system generalized method of moments and find evidence of a clear dynamic nature of the businesses’ sustainability process. The results show that the current levels of the four sustainable development indicators are strongly determined by the levels of these indicators in the two previous years. Our results also show that corporate sustainable development follows a virtuous circle. The relationships across the economic, environmental, and social indicators are bidirectional and positive. Hence, these three sustainability indicators do not compete for available resources. On the contrary, they are tightly interconnected in a firm’s sustainable development processes. Therefore, practitioners and regulators should consider these indicators simultaneously to promote sustainability in businesses and apply long-term sustainability policies. Altogether, our evidence supports the idea that firms can do good by doing well, and they do well by doing good.

## Introduction

The term *sustainability* (or sustainable development) has gained momentum in the last decades. Nowadays sustainable development of a business refers not only to its economic sustainability but also to how sustainable its activities toward the environment and the society are and how good its corporate governance is. Growing concern among shareholders, nongovernmental organizations, environmentalists, regulators, and businesses (Malay, 2021) means that in firms of all sizes environmental, social, and governance (ESG) indicators have become as important as economic indicators for sustainable development (Falco et al., 2021). Hence, a new interest arises in disentangling the way in which different sustainable indicators relate to one other. Accordingly, this study investigates the bidirectional relationship across all four indicators of corporate sustainable development. We develop a general framework researchers, practitioners, and regulators alike can use to explore the feedback effects of the indicators.

Stakeholder theory and resource-based theory posit that firms enjoy economic sustainability (e.g., sustainable financial performance) by addressing the ESG dimensions of sustainability. The underlying reason is that good ESG performance fosters intangible assets such as firm reputation, which, in turn, helps firms to build comparative advantages and improve financial performance. Nadeem et al. (2021) show that resources such as organizational capital are critical for a firm’s environmental performance, such as environmental innovation. In addition, slack resource theory postulates that a firm’s financial resources play an important role in addressing ESG dimensions of sustainability. Together, these theories predict a bidirectional relationship between the indicators of corporate sustainable development.

Whereas underlying theories point to bidirectionality in the relations, studies to date have mainly focused on two or more indicators in a single directional relationship, with most studies examining the relationship between environmental, social or governance, and economic dimensions (e.g., Orlitzky et al., 2003; Tanin et al., 2019; Waddock & Graves, 1997) and very few investigating the reverse (Preston & O’Bannon, 1997; Surroca et al., 2010). Although Khan and Hou (2021) recently reported a mix of bi- and unidirectional relations between the socioeconomic and environmental indicators of sustainable development, a more complete picture of the relationship between a wider range of indicators is still lacking in the literature.

Prior studies also fail to account for the dynamism in a firm’s sustainability process as previous research mainly consider contemporaneous relationships (e.g., Callan & Thomas, 2009). However, the indicators have their own dynamics (Callan & Thomas, 2014; Ng et al., 2020; Waddock & Graves, 1997). Therefore, it is imperative to take into account the full dynamic of each indicator to capture the true relationship between the indicators. This research helps to fill this important gap in the literature and offers a comprehensive analysis of the relationships between the different sustainable indicators.

Finally, except for Ng et al. (2020), prior studies do not consider the potential for unobserved heterogeneity and endogeneity of the independent variables. Failing to address these issues can bias the estimation results, and the model estimated will not display the true relation between the sustainability indicators. Therefore, we use the generalized method of moments (GMM) estimator, which is robust to the existence of unobservable heterogeneity while controlling for endogeneity of the independent variables. More important, the GMM estimator takes into account the dynamic completeness (Wintoki et al., 2012). Thus, we were able to disentangle the true relationship among the four indicators of corporate sustainable development.

We use economic, environmental, social, and governance performance information on 734 U.S. firms from 2004 through 2016 to investigate the bidirectional nature of these four indicators. Constructing the indicators requires a lot of time and money (Lee & Kim, 2021), as well as an availability of data such as corporate performance reports, media coverage, and so on. Consequently, we focus on U.S. companies due to the availability of complete and quality data.

This study contributes to the literature in several ways. First, we consider the bidirectional nature of the relationship between environmental, social, economic and governance indicators, which most studies to date do not address. Second, we use industry-adjusted performance indicators to control the industry effect in all estimations; as a result, our findings are relevant across industries. Third, we help to fill the gap in prior research, which does not fully address the dynamics of the sustainability process. Hence, we study the specification of each model and select the correct specification of each model with the suitable order of dynamics. Fourth, unlike most prior research, we account for heterogeneity and endogeneity issues and thus provide unbiased results. Finally, prior research about the role played by firms’ characteristics in promoting sustainability lacks consensus. Modeling sustainability as a system enables us to investigate the true relative importance of a firm’s characteristics for sustainable development.

The remainder of this study proceeds as follows. Section 2 discusses the theoretical background and develops hypotheses. Section 3 describes the data, variables, and methodology. Section 4 reports the results and discusses the findings. Finally, Sect. 5 provides our conclusions.

## Corporate Sustainable Development Indicators: Hypotheses

The relationship between social/environmental/governance responsibility and corporate financial performance has been widely studied over the last four decades without the research reaching any clear consensus. Concerns over the social dimension of the company date from the 1950s when Levitt (1958) wrote about the dangers of social responsibility and his controversial point of view about the damages that this kind of “sentimentalism” causes in businesses. Friedman (1970) famously stated “The business of business is business,” which called into question the role of social practices in the search for profits. Subsequent research founds that the cost of being socially and/or environmentally responsible outweighs the potential benefits, thus leading to the conclusion that social and environmental commitments can result in lower firm value—in other words, it does not pay to be good (e.g., Fisher-Vanden & Thorburn, 2011; Makni et al., 2009; Walley & Whitehead, 1994).

Despite these findings, the most predominant theoretical and empirical approach points to a positive association between corporate social/environmental responsibility and financial performance. Arguments supporting this positive association usually find their roots in the stakeholder theory (Freeman, 1984) and the resource-based view (RBV) of the firm (Barney, 1991; Wernerfelt, 1984).

According to stakeholder theory, meeting the needs of the various stakeholder groups is instrumental in achieving improved financial performance. Instrumental stakeholder theory posits that managers can increase efficiency by attending to and balancing the claims of the different stakeholders (Freeman & Evan, 1990). Furthermore, a trusting and cooperative relationship between a firm’s management and stakeholder groups reduces managers’ opportunism and gives the firm a competitive advantage (Hill & Jones, 1992; Jones, 1995). Thus, by successfully attending to stakeholders’ demands, firms can improve their reputations, build and maintain long-term relationships with suppliers and customers, and foster efficiency (Hillman & Keim, 2001; Lankoski, 2008).

Within the context of this theory, stakeholders’ increasing concern about the environmental and social consequences of a firm’s activities are relevant. Good management theory suggests that certain altruistic behaviors such as social and environmental responsiveness are positively associated with productivity—in other words, firms “do well by doing good” (Waddock & Graves, 1997). Previous research that contributes to the stakeholder theory shows that environmental commitments are instrumental to stakeholder management (e.g., Buysse & Verbeke, 2003; Pucheta-Martínez et al., 2020). Moreover, addressing sustainable development through corporate policies is increasingly important (Lee & Kim, 2021) due to the positive link between social and environmental indicators at a business level and the corresponding indicators at a macro level (Malay, 2021).

The RBV highlights a firm’s valuable and unique resources and capabilities as the key sources of sustainable competitive advantages. As the importance of social and ecological concerns increased, the RBV required reformulation to connect social and environmental challenges to a firm’s resources. Hart (1995) integrated the natural environment into the mainstream RBV and gave rise to the natural-resource-based view of the firm. According to Hart, strategy and competitive advantages should be rooted in capabilities that facilitate environmentally sustainable economic activities. Within the framework of this theory, social and environmental challenges may lead the firm to develop intangible resources and capabilities related to innovation, human capital, reputation, and culture, which are, in turn, sources of competitive advantages leading to superior financial performance (Russo & Fouts, 1997; Sharma & Vredenburg, 1998; Surroca et al., 2010).

After decades of research, an exhaustive list of studies addresses the relationship between corporate ESG performance and corporate financial performance. Many previous studies use different multidimensional metrics of corporate responsiveness that include social, environmental, and governance concerns. However, the results are mixed. Several meta-analyses (e.g., Busch & Friede, 2018) concluded that social and environmental responsiveness positively affects financial performance. Other studies find differences in the role of ESG in explaining corporate financial performance; namely, social responsibility is a driver of financial performance whereas environmental and governance performance do not affect financial performance (Ahmad et al., 2021; Tanin et al., 2019).

Other research—albeit, more limited—investigates the relationship between corporate governance and social/environmental performance. This strand of the literature focuses on the implications of certain corporate governance structures for a firm’s social performance. This evidence is consistent with the argument that outsider influence in corporate governance, such as outside directors and board diversity, promotes social responsiveness (Bear et al., 2010; Johnson & Greening, 1999; Webb, 2004). Prior research also points to a positive association between institutional ownership and social performance (Graves & Waddock, 1994; Johnson & Greening, 1999). Nonetheless, contradictory results indicate that strong corporate governance, in terms of ownership, financial transparency, and board structure, lead to poor social and environmental performance (e.g., McGuire et al., 2012).

Although most of the research on the relationship between social/environmental responsibility and financial performance is focused on the causal link from the former to the latter, there is a second strand of literature which proposes the reverse direction of this relationship. Specifically, slack resource theory (McGuire et al., 1988) suggests that firms with slack resources potentially available from superior financial performance invest more in socially responsible activities. Supporting this view, there is evidence of a positive effect of a firm’s financial performance on its social/environmental responsibility (Waddock & Graves, 1997 and, more recently, Pucheta‐Martínez et al., 2020b).

Waddock and Graves (1997) also found that financial performance depends on good social performance. Thus, they posited the existence of a virtuous circle in which social performance is both the cause and the consequence of financial performance. However, no consensus exists in the literature on the existence of a virtuous circle. Some evidence, including the meta-analysis by Busch and Friede (2018), corroborated a synergistic relationship between social/environmental responsibility and financial performance (e.g., Preston & O’Bannon, 1997; Surroca et al., 2010). However, other results suggested that the link between ESG and financial performance is asymmetrical; that is, ESG performance significantly affects financial performance whereas the opposite causality is nonsignificant (Callan & Thomas, 2014).

Despite the vast number of studies on the relationship between corporate social, environmental, governance, and financial performance, they offer only partial analyses, and none disentangle the bilateral and reciprocal effect that each type of sustainable performance has on the others. According to Hart (1995), sustainable development, by its very definition, is not restricted to environmental concerns but also involves economic and social concerns. Hence, a more complete analysis is needed to disentangle the relationship that exists between the different indicators involved in a firm’s sustainability. To fill this gap, we draw from the premises in Strezov et al. (2017) and Ukko et al. (2019) who have dealt with three sustainable development dimensions—social, environmental, and economic—and we extend this approach by integrating the remaining dimension of sustainability: governance (Ng et al., 2020; Qureshi et al., 2020; Baraibar‐Diez et al., 2019; Tanin et al., 2019; Whitelock, 2019).

**Fig. 1:**
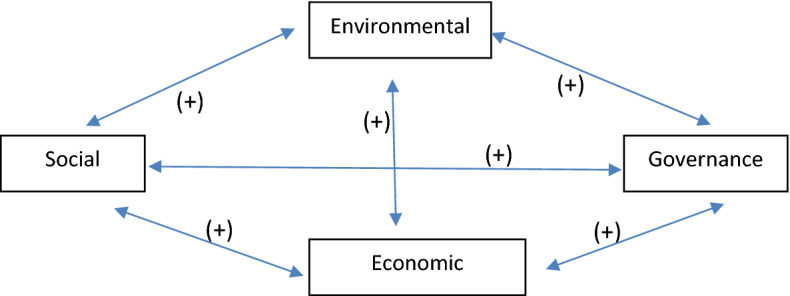
Synergic relationship between the four sustainability indicators

Built upon the notion of a virtuous circle within a firm’s sustainability, we expect that an increase in one of the sustainable development indicators (social, environmental, economic, and governance) leads to increases in the remaining indicators. This expectation relies on the supposition that a firm’s different sustainability policies, rather than competing for resources, may be the source of new resources and capabilities that allow the firm to engage in more sustainable commitments. Within this theoretical framework, and referring to Waddock and Graves (1997), we posit that firms do well by doing good and that doing well is a premise for a firm to do good. Figure 1 shows the synergic relationship between the four sustainability indicators.

To test the existence of this virtuous circle, we propose the following hypotheses:

### Hypothesis 1

There is a positive and bidirectional relationship between a firm’s economic and environmental performance.

### Hypothesis 2

There is a positive and bidirectional relationship between a firm’s economic and social performance.

### Hypothesis 3

There is a positive and bidirectional relationship between a firm’s economic performance and governance.

### Hypothesis 4

There is a positive and bidirectional relationship between a firm’s environmental and social performance.

### Hypothesis 5

There is a positive and bidirectional relationship between a firm’s environmental performance and governance.

### Hypothesis 6

There is a positive and bidirectional relationship between a firm’s social performance and governance.

## Data and Methodology

### Data Sources and Sample

To test our hypotheses, we employ two types of information. First, we use the Worldscope database to obtain information on the financial statements of U.S. firms. Second, we use the Thomson Reuters Refinitiv to obtain companies’ environmental, social, and governance indicators.

To control for unobservable heterogeneity and endogeneity (see Sect. 3.3), we constructed a data panel of U.S. companies for 2004 to 2016. However, we lose the first two years because the correct specifications lead to a dynamic model order 2, as we highlight in the following section. Table 1 provides the distribution of the sample by year.

**Table 1:** Sample breakdown by year

Year	*n*	%
2004	174	3.00
2005	224	3.86
2006	244	4.20
2007	293	5.05
2008	413	7.12
2009	498	8.58
2010	565	9.74
2011	604	10.41
2012	599	10.32
2013	584	10.06
2014	580	9.99
2015	573	9.87
2016	452	7.79
Total	5803	100

The final sample contains 734 U.S. listed companies and 5803 firm-year observations. We only consider companies for which at least five consecutive years of data are available. This requirement is necessary to test for the absence of second-order serial correlation because our estimation method, GMM, is based on this assumption. We exclude financial, insurance, and utilities sectors (two-digit Standard Industrial Classification codes 49 and 60). Following Duchin (2010), we do not exclude industrial firms with businesses in the financial sector because doing so would eliminate from the sample many large conglomerates that maintain a finance division.

### Variables and Models

Given that our aim is to study the relationship between the economic, environmental, social and governance indicators, we build four models. The dependent variable in each model is one of the indicators, and the remaining indicators enter the model as explanatory variables. Consequently, we propose the following models to test our hypotheses:

1 $${ECD}_{it}= {\upbeta }_{0}+{\upbeta }_{1}{ECD}_{it-1}+{{\upbeta }_{2}{ECD}_{it-2}+{\upbeta }_{3}ENVD}_{it}+{\upbeta }_{4}{SD}_{it}+{\upbeta }_{5}{GD}_{it}+{\upbeta }_{6}{SIZE}_{it}+{\upbeta }_{7}{LEV}_{it}+{\upbeta }_{8}{GROWTH}_{it}+{\upbeta }_{9}{CF}_{it}+{\upbeta }_{10}{INTANG}_{it}+{\upeta }_{\mathrm{i}}+{d}_{t}+{\upvarepsilon }_{it}$$

2 $${ENVD}_{it}= {\mathrm{\alpha }}_{0}+{\mathrm{\alpha }}_{1}{ENVD}_{it-1}+{{\mathrm{\alpha }}_{2}{ENVD}_{it-2}+{\mathrm{\alpha }}_{3}ECD}_{it}+{\mathrm{\alpha }}_{4}{SD}_{it}+{\mathrm{\alpha }}_{5}{GD}_{it}+{\mathrm{\alpha }}_{6}{SIZE}_{it}+{\mathrm{\alpha }}_{7}{LEV}_{it}+{\mathrm{\alpha }}_{8}{GROWTH}_{it}+{\mathrm{\alpha }}_{9}{CF}_{it}+{\mathrm{\alpha }}_{10}IN{TANG}_{it}+ {\upeta }_{i}+{d}_{t}+{\upvarepsilon }_{it}$$

3 $${SD}_{it}= {\mathrm{\varphi }}_{0}+{\mathrm{\varphi }}_{1}{SD}_{it-1}+{\mathrm{\varphi }}_{2}{SD}_{it-2}+{\mathrm{\varphi }}_{3}{ENVD}_{it}+{\mathrm{\varphi }}_{4}{ECD}_{it}+{\mathrm{\varphi }}_{5}{GD}_{it}+{\mathrm{\varphi }}_{6}{SIZE}_{it}+{\mathrm{\varphi }}_{7}{LEV}_{it}+{\mathrm{\varphi }}_{8}{GROWTH}_{it}+{\mathrm{\varphi }}_{9}{CF}_{it}+{\mathrm{\varphi }}_{10}{INTANG}_{it}+{\upeta }_{i}+{d}_{t}+{\varepsilon }_{it}$$

4 $${GD}_{it}= {\upomega }_{0}+{\upomega }_{1}{GD}_{it-1}+{\upomega }_{2}{GD}_{it-2}+{\upomega }_{3}{ENVD}_{it}+{\upomega }_{4}{ECD}_{it}+{\upomega }_{5}{SD}_{it}+ {\upomega }_{6}{SIZE}_{it}+{\upomega }_{7}{LEV}_{it}+{\upomega }_{8}{GROWTH}_{it}+{\upomega }_{9}{CF}_{it}+{\upomega }_{10}{INTANG}_{it}+ {\eta }_{i}+{d}_{t}+{\upvarepsilon }_{it}$$

where ECD_it_ denotes the economic indicator as measured by Client Loyalty, Performance, and Shareholder Loyalty. It measures “a company's capacity to generate sustainable growth and a high return on investment through the efficient use of all its resources. It is a reflection of a company's overall financial health and its ability to generate long term shareholder value through its use of best management practices” (Thomson Reuters, 2017). ENVD_it_ is the firm’s environmental indicator which measures “a company's impact on living and non‐living natural systems, including the air, land and water, as well as complete ecosystems. It reflects how well a company uses best management practices to avoid environmental risks and capitalize on environmental opportunities in order to generate long term shareholder value” (Thomson Reuters, 2018). It covers a firm’s resource use, emissions, and innovation. SD_it_ is the firm’s social indicator, and it measures “a company's capacity to generate trust and loyalty with its workforce, customers, and society, through its use of best management practices. It is a reflection of the company's reputation and the health of its license to operate, which are key factors in determining its ability to generate long term shareholder value” (Thomson Reuters, 2018). It considers a firm’s social actions towards workforce, human rights, community, and product responsibility. GD_it_ is the firm’s governance indicators which measures “a company's systems and processes, which ensure that its board members and executives act in the best interests of its long-term shareholders. It reflects a company's capacity, through its use of best management practices, to direct and control its rights and responsibilities through the creation of incentives, as well as checks and balances in order to generate long term shareholder value” (Thomson Reuters, 2018). It concerns management, shareholders, and the firm’s corporate social responsibility (CSR) strategy. As pointed out by Greco et al. (2018) and Cabello et al. (2021), composite indicators such as the ones used in this study are recommended to correctly process and manage a huge amount of information.

Aparicio and Kapelko (2019), among others, found that ESG engagement varies with industries. To capture the engagement (performance) in ESG that is attributable to the individual firm, we use the difference between a firm’s ESG and the industry-year median ESG. We use the Standard Industrial Classification to compute the median and the adjusted firm’s ESG data. All variables used in this study are defined in the appendix.

We also include control variables as suggested by the literature. First, firm size (*SIZE*) is measured as the natural logarithm of firm total assets. Larger firms tend to engage more actively in ESG (Pucheta-Martínez et al., 2020). Second, firm financial risk (*LEVERAGE*), calculated as the ratio of total debt to the sum of market capitalization, total debt, and preferred stock, can influence ESG performance (Callan & Thomas, 2009; Jo & Harjoto, 2011). Third, a firm’s growth potential can influence its ability to invest in ESG activities and thus ESG performance (Berrone et al., 2010; Rees & Rodionova, 2015). We therefore control for growth opportunities (*GROWTH*), measured as the ratio of market capitalization to total assets. Fourth, a firm’s ability to generate cash flow influences investments in ESG activities. Thus, we control for cash flow (*CF*), calculated as the amount of cash flow generated from operations scaled by firm total assets (Callan & Thomas, 2009). Finally, *INTANG* is measured as the intangible assets scaled by total assets (Callan & Thomas, 2014; Marti et al., 2015; Ortas et al., 2019; Yu & Tsai, 2018). Table 2 reports the summary statistics of the variables included in our analyses.

**Table 2:** Summary statistics

	Mean	SD	Min	Max
*ECDit*	− 0.1494	19.9319	− 82.5950	83.4700
*ENVDit*	2.0101	20.0839	− 88.2700	95.2100
*SDit*	0.4006	15.0330	− 58.8550	72.3500
*GDit*	− 0.4381	16.6004	− 73.3700	72.0800
*SIZE*	15.8658	1.3077	12.3217	21.3808
*LEV*	0.1997	0.1668	0.0000	0.9286
*GROWTH*	1.3907	1.4270	0.0048	15.6179
*CF*	0.1070	0.0740	− 0.3725	0.6632
*INTANG*	0.2106	0.2108	0.0000	0.9071

This table presents the main descriptive statistics of the variables used in the analyses. All four dimensions are calculated as the difference between a firm’s performance and the industry-year median performance for the dimension. Variables are defined in Appendix

In all models we control for unobserved heterogeneity by including the individual effect, η_i._. This effect can be modeled in our equations using panel data methodology, which allows us to estimate the parameters of the independent variables without identifying the 657 parameters for the η_i_ (i.e., one per company). The use of panel data requires that we control the effect of macroeconomic variables on each dependent variable. Hence, we include a time-specific effect, d_t_, to control for the macroeconomic influence. Finally, ε_it_ is the random disturbance.

Importantly, to study the relationship between the four indicators, the research process must be based on a well-specified economic model. Previous literature does not consider this aspect. In fact, except for Wintoki et al. (2012), researchers did not pay attention to the specification of the models, and usually started from a static model, or a dynamic order 1 model and thus fail to consider the dynamism of firms’ sustainability process. However, we enter the lags required to ensure the dynamic completeness of the models. The empirical estimation (see Sect. 4) tells us that the model is dynamic order 2. Therefore, our models include one- and two-year lagged values of the dependent variable as a means of capturing the dynamics that we expect to find in sustainability processes.

In fact, it is difficult to conceive that any of the selected independent and control variables is more determinant of the sustainability indicators than their own lags. However, this dynamism has not been considered in previous research, except for Callan and Thomas (2014). They found evidence that a company’s financial and social performance in the present period follows from the two previous periods. But their results may be biased because the use of a static estimator presents two main drawbacks. First, it only captures the interrelation between the dependent variables but does not control for the endogeneity that arises from the control variables, and second, it does not provide a suitable answer to the dynamic specification of the model. For this reason, Ng et al. (2020) use the system GMM to estimate their dynamic model order 1. As we explain next, the system GMM is the most appropriate method to estimate dynamic processes with current realizations of the dependent variable influenced by past realizations. Thus, we take into account the dynamism of the underlying process itself for each indicator.

### Estimation Method

Given that managers make decisions, their individual characteristics influence their decision-making. This effect is unobservable to the researcher and thus does not enter the regressions. If we do not control for this unobservable heterogeneity, we run the risk of obtaining biased results. Consequently, unlike cross-sectional analysis, the panel data methodology allows us to control for unobservable heterogeneity through an individual effect, η_i_.

Moreover, the influence of the dependent variables of our models on some of the right-hand-side variables can be easily recognized. Consider, for instance, a firm’s intangibles. Intangibility may be influenced by a company’s social, environmental, governance, and economic responsiveness if, as literature suggests, these activities improve reputation and increase competitive advantages. These intangibles also affect a firm’s investment opportunities and size.

In our models, the dependent variable enters the other three models as a regressor, which is an important source of endogeneity that can affect the estimation results if an inappropriate method is used. In fact, endogeneity biases the ordinary least squares estimation (Binder et al., 2020; Callan & Thomas, 2014; Yu & Tsai, 2018). Although this bias can be controlled using a simultaneous equation estimator, such as maximum likelihood (Ortas et al., 2019), and two- or three-stage least squares estimators (Callan & Thomas, 2014; Hou, 2019), these estimators would not be consistent with not controlling for endogeneity in the control variables, since this endogeneity biases all the coefficients.

In addition, previous research does not eliminate unobservable heterogeneity. Some studies, such as Cho et al. (2019), Marti et al. (2015), and Tanin et al. (2019) eliminate unobservable heterogeneity by using the within-groups estimator (erroneously referred to in most of the literature as fixed-effects estimator) and the random effects estimator; however, these estimators do not control for endogeneity or dynamism. To address these concerns, we use GMM, which embeds all other instrumental variable estimators. Specifically, we use the system GMM estimator to overcome the weak instruments problem from which the difference GMM estimator suffers. We use the lags from *t* − 2 to *t* − 5 for all the right-hand side variables as instruments for the equations in differences and only one instrument for the equations in levels, as suggested by Blundell and Bond (1998).

This decision is based on the biases reported in the literature (Bond et al., 2001; Hsiao, 1986; Nickell, 1981). We tested for these biases in our model before we made a decision on the chosen estimator. Specifically, we focus on the description of biases and the comparison of models on the lag of the dependent variable for which econometric studies are available that detail the direction of the biases. We start with the ordinary least squares, which is the most basic estimator. Hsiao (1986) finds that the coefficient for the lag of the dependent variable is biased upward in the presence of individual heterogeneity. In fact, Table 3 shows that the ordinary least squares estimator is biased upward for the regression from the four indicators.

**Table 3:** Comparison of estimators across the four dimensions

Estimator/dimension	Economic	Environmental	Social	Governance
Ordinary least squares	0.4281 (0.0141)***	0.7353 (0.0147)***	0.6398 (0.0140)***	0.5564 (0.0144)***
Within-groups	0.1581 (0.0151)***	0.4514 (0.0154)***	0.3339 (0.0149)***	0.2598 (0.0158)***
First-differenced GMM	0.1781 (0.0607)***	0.3296 (0.0595)***	0.2395 (0.0631)***	0.0149 (0.0709)***
System GMM	0.4104 (0.0526)***	0.6024 (0.0455)***	0.5849 (0.0511)***	0.4452 (0.0542)***

This table reports estimation results from the most common estimators for which studies show bias is entered in the lag of the dependent variable. Regression results are from estimating Models 1–4 by using the following estimators: ordinary least squares, within-groups, first-differenced GMM and system GMM. Standard errors are in parentheses

*, **, and *** indicate significance at the 10%, 5%, and 1% level, respectively

The second estimator that we consider is the within-groups estimator, which Nickell (1981) found to be seriously biased downward. Table 3 shows that the coefficient obtained for the four indicators is biased downward with respect to the benchmark (i.e., the system GMM). Finally, Bond et al. (2001) found that the first-differenced GMM estimator is biased downward due to weak instruments, a problem first addressed by Alonso-Borrego and Arellano (1999). The coefficient for the first-differenced GMM estimator is always smaller than the coefficient for the system GMM (see Table 3). As a result, all the estimations discussed in this study rely on the system GMM estimator.

Given that we use the GMM estimator, we check for the potential misspecification of the models. First, we use the Hansen *J* statistic of overidentifying restrictions to test for the absence of correlation between the instruments and the random disturbance. Second, we use the *m*_2_ statistic (Arellano & Bond, 1991) to test for the lack of second-order serial correlation in the first-difference residual. In addition, we use Wald tests to check for the joint significance of the reported coefficients, as well as of the time dummies. Finally, we used Wald tests to check for the joint significance of the control variables.

## Results

This section presents the main results of the estimation of our models. Additionally, two robustness tests are presented in order to check for the correct estimation of the models.

### Main Results

Table 4 presents the regression results that test our hypotheses. Columns 1 to 4 show the results from estimating Models 1 to 4, respectively, which examine the determinants of the economic, environmental, social, and governance indicators of a firm’s sustainability. Our results support the dynamic nature of the process (column 1), indicating that a firm’s current ability to generate sustainable growth and long-term shareholder value is strongly determined by the economic policy followed by the company in the two previous periods. Identical results are obtained when analyzing the other sustainability indicators (columns 2–4). Past realizations of the dependent variables strongly influence current realizations. Hence, our evidence shows that a company’s economic, environmental, social, and governance indicators are dynamic. In other words, we find dynamism in a firm’s sustainability processes.

**Table 4:** Estimation results

	*ECD* (1)	*ENVD* (2)	*SD* (3)	*GD* (4)
Lag1	0.4104 (0.0526)***	0.6024 (0.0455)***	0.5849 (0.0511)***	0.4452 (0.0542)***
Lag2	0.0926 (0.0246)***	0.1693 (0.0350)***	0.13980 (0.0362)***	0.1697 (0.0321)***
*ECDit*		0.0527 (0.0244)**	0.0426 (0.0203)**	0.0366 (0.0306)
*ENVDit*	0.0766 (0.0370)**		0.1101 (0.0222)***	0.0917 (0.0313)***
*SDit*	0.14999 (0.0544)***	0.1411 (.04204)***		0.0544 (0.0434)
*GDit*	0.0459 (0.0416)	0.0499 (0.0288)*	0.03467 (0.0239)	
*SIZE*	0.3081 (0.5424)	0.7771 (0.3560)**	0.25747 (0.2574)	− 0.0836 (0.4166)
*LEV*	2.0964 (4.4114)	− 2.5968 (2.2648)	− 2.2904 (1.9356)	− 0.9300 (2.8940)
*GROWTH*	0.1435 (0.5315)	− 0.9854 (0.3140)***	0.1949 (0.2274)	0.0995 (0.3644)
*CF*	19.9724 (11.5803)*	16.0386 (6.6307)**	− 8.0529 (5.1885)	− 7.3455 (6.9131)
*INTANG*	− 0.1973 (2.8593)	− 1.5418 (2.0284)	− 1.0570 (1.6285)	− 0.3060 (2.2248)
Constant	− 8.6003 (9.5438)	− 11.0066 (6.1396)*	− 3.2773 (4.4276)	1.3459 (6.9599)
z_1_	41.7000 (10)	182.4000 (10)	139.9800 (10)	43.2000 (10)
z_2_	1.2200 (5)	3.9300 (5)	1.2500 (5)	0.2400 (5)
z_3_	2.2400 (10)	2.8000 (10)	4.2500 (10)	2.6200 (10)
m_1_	− 8.0000	− 6.1700	− 6.7200	− 6.9500
m_2_	0.4100	− 1.5200	− 1.2300	− 1.0300
Hansen test of overidentifying restrictions (χ^2^)	386.7500 (357)	394.6000 (357)	378.6900 (357)	380.2000 (357)

This table reports estimation results from dynamic panel models estimated using instrumental-variable GMM estimation. The sample period is from 2004–2016. All four dimensions are calculated as the difference between a firm’s performance and the industry-year median performance for the dimension. Regression results from estimating Models 1–4 by using the system GMM estimator. Standard errors are in parentheses. *, **, and *** indicate significance at the 10%, 5%, and 1% level, respectively. ${z}_{1}$ is a Wald test of the joint significance of the reported coefficients, asymptotically distributed as χ^2^ under the null of no relationship, degrees of freedom in parentheses; ${z}_{2}$ is a Wald test of the joint significance of the control variables, asymptotically distributed as χ^2^ under the null of no relationship, degrees of freedom in parentheses; and ${z}_{3}$ is a Wald test of the joint significance of the time dummies, asymptotically distributed as χ^2^ under the null of no relationship; degrees of freedom are in parentheses. ${m}_{i}$ is a serial correlation test of order *i* using residuals in first differences, asymptotically distributed as N(0,1) under the null of no serial correlation, and Hansen is a test of the overidentifying restrictions, asymptotically distributed as χ^2^ under the null of no correlation between the instruments and the error term; degrees of freedom are in parentheses. Variables are defined in Appendix

Hypothesis 1 is tested by estimating Models 1 and 2, yielding the results which are reported in columns 1 and 2 in Table 4. The coefficient of *ENVD*_*it*_ is positive and significant (column 1). This finding is consistent with stakeholder theory, which suggests that environmental responsiveness helps firms to improve their relationship with stakeholder groups, which, in turn, improves economic performance. This result is also in line with the natural-resource-based view of the firm, which points to environmental responsiveness as a source of competitive advantages and superior financial performance (Hart, 1995). The coefficient of *ECD*_*it*_ is also positive and significant (column 2). This result is consistent with Ortas et al. (2019), Pucheta‐Martínez et al. (2020b) and Khan and Hou (2021) who found that superior financial performance provided slack resources for the company to support environmentally responsible activities. Together, these results confirm Hypothesis 1 that the relationship between a firm’s economic and environmental performance is positive and bidirectional.

Regarding Hypothesis 2, Table 4 (column 1) shows a positive and significant effect of *SD*_*it*_ on *ECD*_*it*_, which is consistent with both stakeholder theory and the RBV approach. As occurs with environmental responsibility, a firm’s social commitment has a positive impact on its sustainable growth and its ability to generate shareholders’ long-term value. In addition, the coefficient of *ECD*_*it*_ is positive and significant (column 3); that is, higher values of economic performance provide slack resources that allow the firm to increase socially responsible activities. This evidence therefore supports Hypothesis 2 that the relationship between a firm’s economic and social performance is positive and bidirectional.

Our results regarding the relationship between the economic and governance indicators (Hypothesis 3) differ from our previous findings. Namely, the coefficient of *GD*_*it*_ is nonsignificant (Table 4, column 1). This result suggests, contrary to Callan and Thomas (2014), that responsible governance practices do not help the firm to improve its ability to grow in a sustainable way. Conca et al. (2020) found similar controversial results. They found a positive relationship between profitability and disclosure practices of strictly environmental and social information and a negative effect between company market value and disclosure practices related to governance. Also, for European companies, Qureshi et al. (2020) found a positive impact of environmental and social disclosures on a firm’s value, whereas governance disclosure did not have any impact.

Our results show no effect of *ECD*_*it*_ on *GD*_*it*_ (Table 4, column 4). This result on *GD*_*it*_ deviates from Ortas et al. (2019), who found that a firm’s economic performance positively affected corporate governance performance. As a result, Hypothesis 3 is rejected. The effectiveness of a corporate governance system depends on the institutional settings within which it finds itself. Leuz et al. (2003), among others, showed that legal systems provide protection to investors outside of the firms and offer governance at the national level. Moreover, Chih et al. (2008) and Leuz et al. (2003) showed that firm-level governance is especially important where an institutional support system is weak. The robust U.S. legal system, with strong investor rights and enforcement, offers a solid level of governance at the institutional level, which may explain the insignificant relationship between *GD* and *ECD* at the firm level.

The estimation results show a positive, significant, and bidirectional relationship between *ENV*_*it*_ and *SD*_*it*_ (Table 4, columns 2 and 3). We thus find support for Hypothesis 4, which posits a positive and bidirectional relationship between a firm’s environmental and social performance. These results suggest that a positive feedback process exists through which environmentally and socially responsible actions do not compete for available resources. That is, these two sustainable indicators, which have been jointly considered as one unique indicator by previous literature, in fact appear to have a beneficial and synergetic relationship. Wood (1991) argued that corporate social responsibility includes the process of social responsiveness (e.g. the environmental assessment, stakeholder management, and issues management) and that the outcomes are measured as social impacts, programs, and policies, which are legitimated within society and the organization, as well as the individuals within the organization. Wood’s principles highlight the intertwined nature of social and environmental activities and suggest that social performance is accompanied by environmental performance.

The results on the relationship between a firm’s environmental and governance practices are similar to our previous findings. *GD*_*it*_ has a positive and significant effect on *ENV*_*it*_ (Table 4, column 2). This result is contrary to McGuire et al. (2012), who found a negative effect of a firm’s corporate governance practices on their environmental performance. This discrepancy may be due to the studies not having captured the full dynamics of the indicators or not fully addressing the endogeneity issue. The influence of *ENV*_*it*_ on *GD*_*it*_ is also positive and strongly significant (column 4). This result is in line with Ortiz-de-Mandojana et al. (2010), who find that firms need a governance structure that supports their environmental ambitions. Both results on *ENV*_*it*_ and *GD*_*it*_ support Hypothesis 5 that the relation between a firm’s environmental performance and its governance is positive and bidirectional. That is, a synergic relationship exists between these two sustainability indicators.

Finally, we test Hypothesis 6, which posits a positive and bidirectional relationship between a firm’s social performance and governance. Table 4 (column 3) shows no effect from *GD*_*it*_ on *SD*_*it*_. This finding is contrary to the evidence in Bear et al (2010), Graves and Waddock (1994), Johnson and Greening (1999), and Webb (2004), who all found a positive effect of strong corporate governance structures on social performance. In addition, the coefficient of *SD*_*it*_ is nonsignificant, which, considered jointly with the former result, does not support Hypothesis 6. That is, no relationship exists between a firm’s socially responsible practices and its governance standards.

Overall, our results corroborate the existence of a virtuous circle in a firm’s sustainability processes based on economic, environmental, and social indicators. Conversely, governance concerns seem to be out of the circle. Our results support the idea that it pays to be good. In other words, a firm’s concerns about the environmental and social consequences of its activity translate into superior economic performance and superior economic performance leads the firm to increase its environmental and social commitment. A similar synergic process exists in the relationship between environmental and social responsiveness: Instead of competing for available resources, both provide reciprocal feedback to one another for sustainable growth. Thus, once a company decides to allocate resources and engage in environmentally and socially responsible activities, it starts down a path toward corporate sustainability. Contrary to the limited findings in the literature, we find that the governance indicator does not have a feedback effect with economic or social indicators. As previously discussed, these findings may be a result of strong governance at the institutional level.

In general terms, our results point to a limited effect from the control variables on the sustainability indicators. Table 4 (column 4) shows that only a firm’s cash flow affects its sustainable economic indicator. As expected, this effect is positive. Size and cash flow positively influence a firm’s environmental responsibility, whereas the effect of investment opportunities is negative (column 2). None of the selected control variables affects a firm’s social and governance indicators (columns 3 and 4).

Table 5 shows that previous studies provide nonconclusive evidence of the effect of firm characteristics on its sustainability indicators. These findings are likely explained by mis-specified models that do not account for the dynamic completeness and estimation methods applied (in most cases, a static method). Within this context, our results on control variables are interesting. Because our model specification and estimation method are more robust, we decipher the true impact of the firm characteristics on the four indicators. This evidence is important as it clarifies the role of the control variables and their ability to explain indicators that are not closely correlated with the characteristics of the company. In fact, our empirical evidence demonstrates that each indicator is most affected by the two closest lag values, followed by the other indicators, and is least affected by firm characteristics.

**Table 5:** Effect of firm characteristics on sustainable indicators

	ECD (1)	ENVD (2)	SD (3)	GD (4)
*SIZE*	(+): Yu and Tsai (2018), Baraibar-Diez et al. (2019), Robaina and Madaleno (2020), This study (−): Marti et al. (2015)	(+): Rees and Rodionova (2015), Ortas et al. (2019), This study (−): Callan and Thomas (2014)	(+): Rees and Rodionova (2015), Ortas et al. (2019) (NS): This study (−): Callan and Thomas (2014)	(+):Rees and Rodionova (2015), Ortas et al. (2019) and Baraibar-Diez et al. (2019) (NS): This study (−): Callan and Thomas (2014)
*LEV*	(NS): This study	(+): Rees and Rodionova (2015) (NS): This study	(+): Rees and Rodionova (2015) (NS): This study	(+):Rees and Rodionova (2015) (NS): This study
*GROWTH*	(+): Yu and Tsai (2018) (NS): This study	(−): Rees and Rodionova (2015), This study	(−): Rees and Rodionova (2015) (NS): This study	(NS): This study
*CF*	(+): Marti et al. (2015), Baraibar-Diez et al. (2019) (NS): This study	(+): Rees and Rodionova (2015), This study	(+): Rees and Rodionova (2015) (NS): This study	(+): Baraibar-Diez et al. (2019) (NS): This study (−): Ortas et al. (2019)
*INTANG*	(NS): This study (−): Callan and Thomas (2014), Marti et al. (2015)	(NS): This study	(NS): This study	(NS): This study

This table summarizes the effect of firm characteristics on sustainable indicators as found in the literature versus the effect found in Table 4. NS stands for not significant. Variables are defined in Appendix

### Robustness

We first highlight the importance of accounting for an appropriate order of the dynamics of the sustainability variables. In the literature, most studies examine contemporaneous effects (e.g., Arora & Dharwadkar, 2011; Callan & Thomas, 2009), and very few accounted for a one-period lagged effect (Waddock & Graves, 1997 and Ng et al., 2020, for example). Our results in Table 4 show that the four sustainability indicators have a r two. That is, the current level of performance on an indicator is highly influenced by its performance in the two most recent past periods. This finding implies that studies that considered only a one-period lag in the analyses may have incompletely captured the dynamic of corporate sustainability processes.

Table 6 reports estimation results when only one lag of the dependent variable enters the models. The four models suffer from second-order correlation in the first-differenced residuals, as indicated by the significant *z*-statistics for Arellano–Bond tests. Thus, an incomplete account of the full dynamic of corporate sustainability processes can give rise to a second-order correlation in the first-differenced residuals and, thus, an inappropriate model specification. In fact, these results make sense, as the information that can be captured by the second lag goes to the error term, which, along with the strong dynamic of the models, gives rise to the second-order correlation.

**Table 6:** Robustness check 1

	*ECD* (1)	*ENVD* (2)	*SD* (3)	*GD* (4)
Lag1	0.4971 (0.0431)***	0.7553 (0.0327)***	0.7011 (0.0375)***	0.6195 (0.0390)***
*ECD*_*it*_		0.0514 (0.0240)**	0.1202 (0.0256)	0.0568 (0.0271)**
*ENVD*_*it*_	0.0805 (0.0393)**		0.0295 (0.0222)***	0.0834 (0.0285)***
*SD*_*it*_	0.0989 (0.0548)*	0.1572 (0.0419)***		0.0468 (0.0418)
*GD*_*it*_	0.0593 (0.0446)	0.0531 (0.0259)**	0.4270 (0.2736)	
*SIZE*	0.4873 (0.5474)	0.9317 (0.3432)***	0.4556 (1.9366)	− 0.0372 (0.4237)
*LEV*	4.2673 (3.8055)	− 1.6938 (2.2233)	0.3156 (0.2349)	− 1.4277 (2.9407)
*GROWTH*	0.4057 (0.4280)	− 0.7740 (0.2359)***	− 5.3134 (4.7026)	− 0.0285 (0.3092)
*CF*	13.0205 (10.2181)	16.4777 (5.9857)***	− 1.2312 (1.7107)	− 7.9319 (6.3099)
*INTANG*	− 0.1181 (3.2321)	− 2.2528 (1.9481)	− 6.6559 (4.5864)	− 1.1646 (2.7813)
Constant	− 10.4239 (9.4177)	− 13.8860 (5.7537)**	− 6.6559 (4.5864)	1.8820 (6.7839)
z_1_	34.4500 (9)	238.8500 (9)	149.5500 (9)	60.4900 (9)
z_2_	0.8600 (5)	4.5600 (5)	1.0500 (5)	0.5200 (5)
z_3_	2.2800 (10)	3.9500 (10)	4.1500 (10)	2.3200 (10)
m_1_	− 11.3400	− 9.6200	− 10.8500	− 11.5600
m_2_	− 3.0800	1.9800	1.3000	3.6600
Hansen test of overidentifying restrictions (χ^2^)	392.4500 (367)	415.3900 (367)	380.5800 (367)	384.7400 (367)

This table reports results from the re-estimation of dynamic panel models in Table 4 without one lag of the dependent variable. All four dimensions are calculated as the difference between a firm’s performance and the industry-year mean performance for the dimension. Regression results from estimating Models 1–4 by using the system GMM estimator. Standard errors are in parentheses. *, **, and *** indicate significance at the 10%, 5%, and 1% level, respectively. ${z}_{1}$ is a Wald test of the joint significance of the reported coefficients, asymptotically distributed as χ^2^ under the null of no relationship, degrees of freedom in parentheses; ${z}_{2}$ is a Wald test of the joint significance of the control variables, asymptotically distributed as χ^2^ under the null of no relationship, degrees of freedom in parentheses; and ${z}_{3}$ is a Wald test of the joint significance of the time dummies, asymptotically distributed as χ^2^ under the null of no relationship; degrees of freedom are in parentheses. ${m}_{i}$ is a serial correlation test of order *i* using residuals in first differences, asymptotically distributed as N(0,1) under the null of no serial correlation, and Hansen is a test of the overidentifying restrictions, asymptotically distributed as χ^2^ under the null of no correlation between the instruments and the error term; degrees of freedom are in parentheses. Variables are defined in Appendix

As a second robustness check, we investigate whether our results remain identical after re-estimating our four models without controlling for the possibly small size of our sample of only 657 companies. Even though we control for within-panel heteroscedasticity, the estimator will yield standard errors that are downward biased if standard errors are not corrected. This downward bias in standard errors may lead to an upward bias in the statistical significance of the coefficient estimates.

Consistent with this assumption, the coefficient values in Table 7 are the same as those in Table 4, but standard errors are relatively smaller in Table 7. This finding suggests a relationship between some indicators that does, in fact, not exist. For example, when we estimate the models with the small sample correction, the governance indicator is not relevant to the economic, environmental, or social indicators (as reported in Table 4). However, when we re-estimate the models without the small sample correction, standard errors are downward biased and coefficients become significant. Thus, Table 7 reports apparent bilateral relations between governance and the three remaining indicators. This result may explain the conflicting results we find here in light of the positive effect of governance found in the literature (Bear et al., 2010; Callan & Thomas, 2014; Johnson & Greening, 1999; Webb, 2004).

**Table 7:** Robustness check 2

	*ECD* (1)	*ENVD* (2)	*SD* (3)	*GD* (4)
Lag1	0.4104 (0.0165)***	0.6024 (0.01090)***	0.5849 (0.0137)***	0.4452 (0.0173)***
Lag2	0.0926 (0.0087)***	0.1693 (0.0089)***	0.1398 (0.0099)***	0.1697 (0.0098)***
*ECD*_*it*_		0.0527 (0.0073)***	0.0426 (0.0068)***	0.0366 (0.0108)***
*ENVD*_*it*_	0.0766 (0.0138)***		0.1101 (0.0060)***	0.0917 (0.0106)***
*SD*_*it*_	0.1499 (0.0211)***	0.1411 (0.0124)***		0.0544 (0.0147)***
*GD*_*it*_	0.0459 (0.0142)***	0.0499 (0.0087)***	0.0347 (0.0082)***	
*SIZE*	0.3081 (0.2076)	0.7771 (0.1191)**	0.2575 (0.0995)**	− 0.0836 (0.1473)
*LEV*	2.0964 (1.5558)	− 2.5968 (0.8176)***	− 2.2904 (0.7160)***	− 0.9300 (0.9859)
*GROWTH*	0.1435 (0.1664)	− 0.9854 (0.0838)***	0.1949 (0.0853)**	0.0995 (0.1209)
*CF*	19.9724 (3.1972)***	16.0386 (1.8060)***	− 8.0529 (1.7650)***	− 7.3455 (2.3537)***
*INTANG*	− 0.1973 (1.1203)	− 1.5418 (0.6985)**	− 1.0570 (0.6373)*	− 0.3060 (0.84907)
Constant	− 8.6003 (3.7140)	− 11.0066 (2.0495)***	− 3.2773 (1.7627)*	1.3459 (2.4830)
z_1_	353.8000 (10)	4133.4700 (10)	1329.4000 (10)	479.4400 (10)
z_2_	15.6400 (5)	61.7400 (5)	9.4600 (5)	2.0500 (5)
z_3_	11.0300 (10)	26.6200 (10)	32.9100 (10)	12.5400 (10)
m_1_	− 12.3200	− 8.6100	− 11.1000	− 11.0600
m_2_	− 0.6000	− 2.0500	− 1.7000	− 1.4500
Hansen test of overidentifying restrictions (χ^2^)	385.7500 (357)	394.6000 (357)	378.6900 (357)	380.2000 (357)

This table reports results from the re-estimation of dynamic panel models in Table 5 without robust standard errors. All the four dimensions are calculated as the difference between a firm’s performance and the industry-year mean performance for the dimension. Regression results from estimating Models 1–4 by using the system GMM estimator. Standard errors are in parentheses. *, **, and *** indicate significance at the 10%, 5%, and 1% level, respectively. ${z}_{1}$ is a Wald test of the joint significance of the reported coefficients, asymptotically distributed as χ^2^ under the null of no relationship, degrees of freedom in parentheses; ${z}_{2}$ is a Wald test of the joint significance of the control variables, asymptotically distributed as χ^2^ under the null of no relationship, degrees of freedom in parentheses; and ${z}_{3}$ is a Wald test of the joint significance of the time dummies, asymptotically distributed as χ^2^ under the null of no relationship; degrees of freedom are in parentheses. ${m}_{i}$ is a serial correlation test of order *i* using residuals in first differences, asymptotically distributed as N(0,1) under the null of no serial correlation, and Hansen is a test of the overidentifying restrictions, asymptotically distributed as χ^2^ under the null of no correlation between the instruments and the error term; degrees of freedom are in parentheses. Variables are defined in Appendix

Both robustness tests show the importance of correctly estimating the models and explain the differences that we find in comparison with the previous literature on the indicators of corporate sustainable development. In sum, the robustness of our results provides significant relevance to the conclusions from our study.

## Conclusions

This study analyzes the relationship between the four sustainability indicators—environmental, social, governance, and economic. We find a virtuous circle in a firm’s sustainable development processes. Unlike previous studies, our approach addresses the endogeneity issue and captures the whole dynamic of the four indicators of a firm’s sustainability. Specifically, the GMM approach enables us to uncover the true relationship across the sustainability indicators.

We find that all four sustainability indicators exhibit a dynamic of order two. This finding suggests that any strategies businesses formulate to address sustainable development take effect with a certain delay. For instance, several years may pass before a new recycling strategy takes full effect. Thus, businesses should take this information into account in the evaluation of their strategies. Moreover, it is very important for practitioners and regulators not only to consider the various indicators simultaneously to promote sustainability in businesses but also to apply long-term policies, as the dynamic nature limits the effect of short-term policies.

Second, we find evidence that it pays to be good. Particularly, addressing environmental and social sustainability issues results in an improvement in a firm’s economic sustainability. For instance, Walmart works closely with its suppliers to address the company’s goal to cut carbon emissions. A close working relationship with the suppliers gives the company the relationships necessary to survive shocks such as the global financial crisis of 2008 and the Covid-19 pandemic in 2020.^1^

Third, our evidence shows that firms not only can do good by doing well, but they can also do well by doing good. The results show a bidirectional relationship between the environmental/social indicators and the economic indicator. This finding suggests that a virtuous circle exists in a firm’s sustainability process. In other words, addressing environmental/social issues enables firms to sustain themselves economically, which eventually enables firms to further address environmental/social issues. This result suggests that businesses that employ these strategies will enjoy the virtuous circle effect over time as the sustainability process sustains itself.

Fourth, the governance indicator is relevant for businesses to address environmental issues. Firms need to make substantial capital investments to address their environmental goals (e.g., the acquisition of modern technology to reduce emissions), and they thus need good governance to oversee these investments. Therefore, a firm’s environmental strategies should go hand in hand with its governance strategies. Furthermore, given that environmental strategies generate positive economic results, the governance indicator also positively contributes to these results. In sum, it also pays to have good governance practices, although in an indirect way.

Finally, we find that firm characteristics do not play an important role in a firm’s sustainability. The only exceptions are that firms with higher levels of cash flow exhibit higher economic and environmental performance and thus have funds available to jumpstart the sustainability process by addressing environmental and social issues. The lack of significance of the remainder of firm-level characteristics has important implications for policymakers, investors, academics, and the businesses themselves. Namely, policymakers should promote sustainability regardless of the firm’s characteristics, which means a kind of democratization of the sustainability processes. In addition, investors and academics should not associate a firm’s characteristics (e.g., firm size, growth, or leverage) with its sustainability process. Finally, if the aim is to generate sustainable development, businesses should focus on formulating and implementing strategies that promote the indicators of sustainability simultaneously.

The finding that governance is less intertwined with the other indicators of sustainability calls for further analysis. We anticipated that this result may be due to strong governance at a national level in the United States. As previously discussed, this finding differs substantially from previous studies carried out in the United States and other institutional contexts. In addition, we base our analyses on U.S. data due to the availability of reliable data for a long interval, which is critical to our methodology. However, Ahmad et al. (2021), Duque-Grisales and Aguilera-Caracuel (2019), and Johnson et al. (2019) showed that the relationship between financial performance and ESG performance differs across countries. Therefore, future research is needed to determine how these relationships vary across countries.

To conclude, this study has important implications for both academics and practitioners. For academics, our findings highlight the importance of taking into consideration the bidirectional nature of the relationship between the indicators of sustainability, which is lacking in most studies to date. For practitioners, this study sheds light on the interactive nature of the various indicators driving sustainable development in businesses. Regulators should therefore consider the various indicators simultaneously to promote corporate sustainable development.

## Appendix

Variable definitions

**Table:**

Variable	Definition
*Main variables*	
Economic performance (*ECD*)	“Economic performance score reflects a company's capacity to generate sustainable growth and a high return on investment through the efficient use of all its resources. It reflected a company's overall financial health and its ability to generate long term shareholder value through its use of best management practices” (Thomson Reuters, 2017)
Environmental performance (*ENVD*)	“Refinitiv's Environment Pillar Score is the weighted average relative rating of a company based on the reported environmental information and the resulting three environmental category scores: resource use, emission, and environmental innovation information reported” (Thomson Reuters, 2018)
Social performance (*SD*)	“Refinitiv's Social Pillar Score is the weighted average relative rating of a company based on the reported social information and the resulting four social category scores: workforce, human rights, community, and product responsibility” (Thomson Reuters, 2018)
Governance performance (*GD*)	“Refinitiv's Governance Pillar Score is the weighted average relative rating of a company based on the reported governance information and the resulting three governance category scores: management, shareholders, and CSR strategy” (Thomson Reuters, 2018)
*Control variables*^*a*^	
Growth (*GROWTH*)	Market capitalization/total assets
Cash flow (*CF*)	Cash flow from operations/total assets
Firm size (*SIZE*)	Natural logarithm of total assets
Intangibility (*INTANG*)	Intangible assets/total assets
Leverage (*LEVERAGE*)	Total debt/(market capitalization + total debt + preferred stock)

^a^Source for control variables is WorldScope.

## Footnotes

1 Reuters reported on August 18, 2020 that Walmart was beaten on profit and posted record online sales during the Covid-19 pandemic in 2020 (https://www.reuters.com/article/us-walmart-results-idUSKCN25E1D7).
